# Technical Factors Affecting Ultrasound Breast Tumor Size as Correlated with Pathological Type

**DOI:** 10.3390/medicina55110713

**Published:** 2019-10-25

**Authors:** Eman Ahmed Shawky Sabek, Hala Taha Salem

**Affiliations:** Health Radiation Research Department, National Center of Radiation Research and Technology, Atomic Energy Authority, 3 Ahmed El-zomor street, Nasr City, P.O. BOX 9621, Cairo 11765, Egypt; dr-halaa@hotmail.com

**Keywords:** breast lesion, ultrasound, tumor sizing, pathology

## Abstract

*Background and Objectives:* Accurate breast tumor sizing is very important in treatment planning; as a result, ultrasound (US) plays an important role in diagnosing breast masses, due to its non-magnified image and its availability. The continuous change in the disease pathogenesis of breast cancer and tremendous advances in US imaging technology require the continuous evaluation of this imaging modality. In this study, our aim was to determine the accuracy of US in measuring the size of breast mass, and if there is an influence of the different pathological types on this accuracy. *Materials and Methods:* This study contained 66 specimens of breast masses that underwent surgical excision and pathological examination of the resected masses; the mean difference between the size taken by US and the size taken by pathology was calculated to the patients as a whole and for each tumor type in this study. *Results:* The result was that US underestimates the size of the tumor by 0.5 cm for all pathological types, and the US size is in agreement with the pathology size. *Conclusions:* US is an accurate method in measuring breast lesions with a degree of underestimation that may be related to many factors such as the tumor type, size, and margins. Complementary MRI is recommended in case of ILC and architectural distortion.

## 1. Introduction

Breast cancer is the most frequently diagnosed cancer and the leading cause of cancer death among females worldwide [1].

The exact pre-therapeutic tumor size is very important in both the treatment plan and decision making [1]. Conservative management, as lumpectomy or quadrantectomy, is made possible by continuous improvement of the imaging modalities, as it significantly depends on the relationship of the tumor to the breast size [2].

Breast tumor size can be evaluated by clinical examination, mammography, ultrasound (US), and magnetic resonance imaging (MRI). They act in a complementary manner, as each one has its points of strength and weakness. 

Mammography has always been considered the gold standard for diagnosis, its main importance is the detection of microcalcification; however, its accuracy is largely affected by breast density [3]. 

In recent years, high-resolution US and MRI have been strongly incorporated in managing breast lesions.

US is the most available modality; nearly all medical centers contain US machines, in contrast to MRI, which is not that available; many patients must be referred to larger centers to perform MRI. US is a bedside test that does not need contrast injection or specific timing for proper results. Another important factor is cost effectiveness; US will not exhaust the health insurance programs, which is a major issue nowadays. Moreover, the relatively short time of scan, absence of radiation hazards, and the possibility for image-guided biopsy are more added benefits of US.

Although MRI has a high sensitivity, it has low specificity for breast lesions, and it also tends to overestimate lesion size. The main importance of MRI is the detection of multicentricity and the extent of infiltration. Many factors hinder the use of MRI as a basic management technique for breast lesions: its lesser availability, lower specificity, and the expensiveness are the most important [4].

The difficulty of using MRI in every case makes it necessary to frequently re-evaluate the accuracy of US and enhance its role in diagnosing breast masses and accurately measuring breast tumor size, exploiting its benefits, and reserving MRI only for those cases in which the US fails to give accurate measurement and precise extension of the disease.

## 2. Aim of the Work

The purpose is to study the agreement between US and pathology in determining the size of the breast lesion, evaluate the US as a decision-making tool in the pre-therapeutic assessment of breast lesions, and study the technical factors related to the tumour type that affect size measurement.

## 3. Patients and Methods

The data for this retrospective study were obtained from our institute database records and include female patients who had breast lesions and underwent operative excision of the tumor within one week of US examination followed by histopathological examination of the specimen during the period from 2017 to 2019. (As the study was a retrospective, the ethical code was not needed, the data came from a database that is available and follow the protocol of our department). All these patients had been scanned by either one of two physicians who had at least six years of experience in breast imaging at that time. Patients scanned by other physicians have been excluded, as well as those who have been treated with radio or chemotherapy. 

All procedures followed were in accordance with the ethical standards of the responsible committee on human experimentation (institutional and national) and with the Helsinki Declaration of 1964 and later versions.

### 3.1. Image Procedures and Analysis

All the patients were examined using a GE machine LOGIQ P6 with a linear probe 3.42–13 MHz; the patient was in both supine and sitting position with the arm of the examined side put over the head to expose axilla. The examination was carried out both radially and anti-radially to cover all ducto-lobular components, the mass is further analyzed regarding the site, the longest diameter of the mass in cm, echogenicity, shadowing, calcification, and borders. If there is an echogenic halo surrounding the tumor (i.e., desmoplastic reaction), it will be included in the measurement. Then, color Doppler is used to assess the vascularity of the mass. In our study, the largest mass dimension is taken and compared with the largest size taken by the post-operative pathology report; the result of pathology is divided into pathological categories including invasive ductal carcinoma (IDC), ductal carcinoma in-situ (DCIS), invasive lobular carcinoma (ILC), and others (tumors had to be excised for some reason and proved to be fibrocystic disease, ductal hyperplasia, medullary carcinoma, hemorrhage, and fibroadenoma). 

### 3.2. Pathological Procedure

Pathological data were obtained from the pathological report of gross specimen. All specimens were received and fixed before gross cutting. An experienced pathologist cut the largest tumor section, usually in the center of the tumor mass, and measured the maximal tumor diameter using a straight metal ruler.

### 3.3. Statistical Analysis

Data were analyzed using Statistical Package for Social Science (SPSS) version 21.0. Quantitative data were expressed as mean ± standard deviation (SD). Qualitative data were expressed as frequency and percentage. Our null hypothesis states that there is no difference between the US and the pathology measurements. A one-sample t-test was run to determine whether to accept or reject this hypothesis. A *p*-value < 0.05 was considered significant, and a *p*-value < 0.01 was considered highly significant. 

The required sample size for this method comparison study was done by MedCalc for Windows, version 15.0 (MedCalc Software, Ostend, Belgium). The calculation was based on the results of a pilot study that revealed a mean difference of 3.806 cm and an SD of 1.052 between the pathology and US measurements of tumor sizes, with the power of the study set at 90% and alpha at 0.05. It was estimated that a sample size of 95 lesions would be enough to accept or reject our hypothesis. In addition, the same later software was used to create an Altman–Bland plot to represent the mean difference and the limits of agreement (LoA), which are defined as the mean difference ± 1.96 SD of differences and the maximum allowed difference (Δ), which was set at ± 2.8 cm obtained from the same pilot study. If these limits of agreement do not exceed the maximum allowed difference between methods, the two methods are considered to be in agreement, and may be used interchangeably.

## 4. Results

This retrospective study includes 85 patients that underwent surgical excision of 95 breast lesions sent for histopathological examination within one week of the US examination.

The patients’ age, frequency of the different tumor types, and laterality are shown in Table 1. This table shows that the invasive ductal carcinoma (IDC) is the most prevalent tumor, representing 53.7% of the total number of lesions. 

The results of the one-sample *t*-test show that the differences between the measurements of the histopathological examination and the US measurements are significant regarding the whole population and with each tumor type, with all *p*-values less than 0.05. Table 2 represents the mean differences between pathology and US measurements in all tumor types and the 95% confidence intervals of these differences and the *p*-values. In addition, the same table shows that the highest mean difference was shown in the invasive lobular carcinoma lesions: 2.43 cm. 

**Figure 1:**
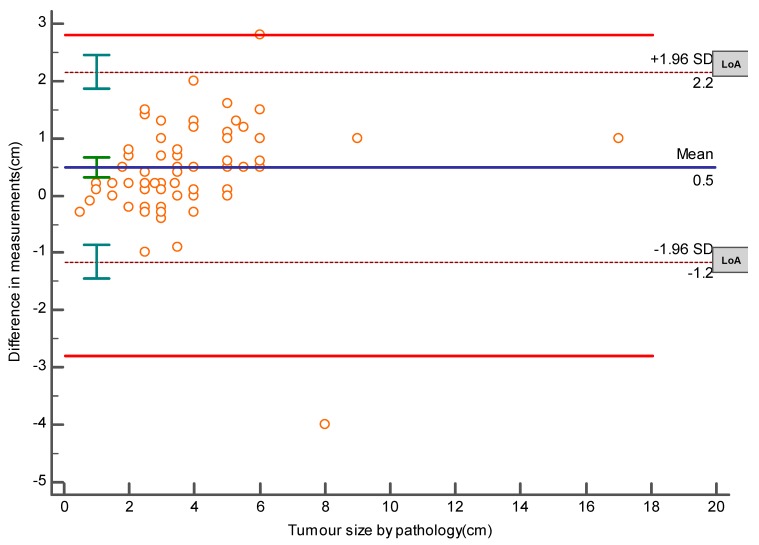
A Bland–Altman plot representing a scatter diagram of the differences plotted against the pathology measurements. It represents the mean difference (blue line) = 0.5 cm, the levels of agreement (LoA) represented by the dashed lines, and the maximum allowed difference represented by the red lines. The upper LoA is equal to 2.2 cm, while the lower LoA equals to −1.2 cm. Error bars represent 95% confidence intervals.

The Bland–Altman plot (Bland and Altman, 1986 and 1999), or difference plot, is a graphical method to compare two measurement techniques. The plot demonstrates the mean difference between the histopathological and the US measurements, which is 0.5 cm, the upper and lower limits of agreement (−1.2, 2.2 cm), and demonstrates that these limits do not exceed the set maximum allowed difference between methods, indicating that the two methods are considered to be in agreement, and may be used interchangeably [4], as shown in Figure 1.

## 5. Discussion

Accurate tumor sizing is very fundamental for treatment planning [2], because it can influence the therapeutic decision and outcome. Ultrasound is an available bedside test with a short time of scan, no preparation needed, and the ability to diagnose lymph nodes and take guided biopsy samples. All these factors facilitate US being a very important tool in diagnosing breast lesions. In our study, the ultrasound underestimates the pathology dimensions with a mean difference of 0.5 cm in the overall sample. Pritt et al. showed that US consistently underestimates pathologic size with an overall difference of 0.35 cm [1]; Gruber et al. showed marked underestimation reaching 0.8 cm [2]. Other authors confirmed the underestimation of the size using ultrasound [5,6].

Underestimation was attributed to many factors; one of them is the unclear margin of the tumor in case of an intraductal in situ component [5]. However, in our study, the largest mean difference in measurements was found in the ILC pathology, which was likely due to the infiltrative type of the lesion [7,8].

Another important factor is the difference in edge perception that will be of great value especially for a small tumor size; elastography technique is used to decrease the difference between the operators, which was not used in those patients. Another edge problem is the echogenic halo around the tumor that, according to previous literature, must be included in the measurement. [9,10], as a retrospective study; one cannot be sure that all echogenic halos were included. Posterior acoustic shadowing is another problem when it hides the posterior edge of the tumor, especially in small tumors [11,12]. In this study, six lesions appeared as areas of architectural distortion during US examination. However, a mass was detected by pathology; these were three IDC cases, one DCIS, one intraductal hyperplasia, and a case of hemorrhage.

In large tumors, in which the size is too large to be taken in one view, there will be an increase in the difference between ultrasonic and pathology measurements. In our study, there were two large lesions that exceeded the width of the transducer; two IDCs were 12 cm by US and 10.5 cm by pathology; the other was 16 by US, and by pathology, it was shown to be 17 cm. In these circumstances, a panoramic view is recommended to decrease the fallacies [7]. One of the most useful tricks that can be used when the panoramic view is not available is the use of an abdominal transducer, as it helps to detect the size accurately. Bosch et al. linked the degree of underestimation to the size of the tumor, especially if the tumor size exceeds the width of the transducer [7]. In this study, the mean differences between the pathology and US measurements differ among the various histologic subtypes; this difference was statistically significant in cases of IDC (a mean difference of 0.3 cm) and for DCIS with or without an invasive component (a mean difference of 0.74 cm). The latter was possibly due to the intraductal component of the tumor, which led to blurring of the edges. In addition, the differences were statistically significant in cases of ILC (mean difference was 2.43 cm), which was the largest among the four pathological categories; it is likely that this was possibly due to the small sample size in this study and the infiltrative type of the lesion. The mean difference in measurements in other tumor types (0.61 cm) may be due to the inclusion of multiple pathological types that produce architectural distortion and irregular pathology such as hemorrhage, ductal hyperplasia, and fibrocystic disease. Pritt et al. reported that the underestimation mean difference was 0.25 cm for ductal carcinoma in situ, 0.3 cm for mixed pattern, and 0.75 cm for lobular carcinoma in situ [1]. Finlayson and McDermott showed that the mean difference is 0.33 cm in case of an infiltrating ductal carcinoma [13], Skaane and Skjorten stated that invasive lobular carcinomas larger than 3 cm were heavily underestimated by ultrasound [14]. Many studies like Cortadellas et al., Moon et al. and Varga et al. compared ultrasonography with mammographic examination and concluded that ultrasonography provided the most precise preoperative size assessment, and US shown to have the highest correlation coefficient and the lowest standard deviation [15,16,17]. 

Despite underestimation done by US, the Bland–Altman plot, which is a graphical method to compare two measurements techniques, demonstrates that the two methods agree and accordingly we can rely on its result.

Efforts must be directed to enhance the usefulness of US for two major concerns nowadays. One of them that there is no radiation exposure and hence it can be repeated whenever needed without radiation hazards. The second is cost effectiveness, which will increase the compliance of health insurance programs.

Regarding the drawbacks of US, one of them that it is operator dependent. In our study, we largely omitted this factor by excluding examiners who had less than five years’ experience; however, this is not the case in real practice. Continuous improvement of operator skills that make them aware of factors affecting tumor size estimation and how to overcome obstacles will further enhance the role of US.

Of course, US cannot be taken separately when diagnosing breast lesions, and we must consider all methods for pre-surgical assessment, using clinical examination, mammography, US, and MRI as complementary tests, and knowing the strengths and weaknesses of each test, in order to plan the best treatment.

Lastly, regarding the histopathology that was done by the staff of the histopathology department, one should take in consideration the interobserver variability that may range from 4 to 32 mm [18]. Another source of error is tumor shrinkage during fixation and difficulty defining tumor margins, particularly for large tumors when only macroscopic measurements are possible [19].

It is well known that deviation from actual results is inevitable, even at the histopathological level as described, knowing the average deviation in each test will help us to overcome errors and ensure satisfactory benefit.

## 6. Conclusions

There is some difference between US measurements and pathology owing to the pathology type, size, and margins of the lesion. However, the two methods agree, and US can be used accurately in sizing breast lesions as a preoperative assessment tool. We recommend complementary MRI in case of ILC, and architectural distortion for better treatment outcomes.

We also recommend the inclusion of the histological subtype for planning surgery in order to estimate the safety margin accurately.

The continuous training of doctors is a must, making them aware of technical factors that will affect the accurate size estimation for better results.

## Figures and Tables

**Table 1:** Descriptive statistics for age, tumor type, and lesion side. (IDC = Invasive duct carcinoma, DCIS = ductal carcinoma in situ, ILC = invasive lobular carcinoma, Others = another lesions pathology).

Age		47.21 ± 12.03
Lesion Type	IDC	51 (53.7)
	DCIS	20 (21.1)
	Others	17 (17.9)
	ILC	7 (7.3)
Lesion side	Right	51 (53.7)
	Left	34 (35.8)
	Bilateral	10 (10.5)

Data are expressed as mean ± SD or number (percentage).

**Table 2:** Mean differences (in cm) between pathology and ultrasound (US) measurements in all tumor types. IDC = invasive duct carcinoma, DCIS = ductal carcinoma in situ, ILC = invasive lobular carcinoma, Others = another lesions pathology.

	Size by Pathology	Size by US	Paired Difference			*p* Value
			Mean Difference	95% Confidence Interval of the Difference		
				Lower	Upper	
All patients	3.97 ± 2.53	3.47 ± 2.49	0.50 ± 0.84	0.32	0.67	<0.001
IDC group	4.29 ± 3.05	3.99 ± 3.03	0.30 ± 0.84	0.06	0.54	0.014
ILC	3.86 ± 0.89	1.43 ± 1.78	2.43 ± 1.52	1.02	3.83	0.006
DCIS	3.58 ± 1.56	2.84 ± 1.06	0.74 ± 0.91	0.31	1.16	0.002
Others	3.55 ± 2.21	2.94 ± 2.25	0.61 ± 0.81	0.19	1.02	0.007

Data are expressed as mean ± SD; 95% confidence intervals of the means are mentioned as (lower, upper bounds); *p*-value < 0.05 is considered significant.
